# Performance of Quantitative PCR to Distinguish *Pneumocystis jirovecii* Pneumonia From Colonisation in Immunocompromised Patients

**DOI:** 10.1111/myc.70120

**Published:** 2025-10-16

**Authors:** Sara Cederwall, Erik Ottander, David Björkhem, Karl Oldberg, Lisa I. Påhlman

**Affiliations:** ^1^ Department of Clinical Sciences Lund, Division of Infection Medicine Lund University Lund Sweden; ^2^ Division of Infectious Diseases Skåne University Hospital Lund Sweden; ^3^ Wallenberg Centre for Molecular Medicine Lund University Lund Sweden; ^4^ Clinical Microbiology Office for Medical Services, Region Skåne Lund Sweden

**Keywords:** colonisation, diagnostics, *pneumocystis jirovecii*, pneumonia, quantitative PCR

## Abstract

**Background:**

*Pneumocystis jirovecii* pneumonia (PCP) is a severe opportunistic infection affecting immunocompromised patients. Quantitative polymerase chain reaction (qPCR) is widely used for the detection of *P. jirovecii* in respiratory samples. However, the diagnosis of PCP remains challenging and the high prevalence of *P. jirovecii* airway colonisation complicates the interpretation of positive results. The aim of this study was to assess the utility of *P. jirovecii* PCR Quantification Cycle (Cq) values in differentiating between PCP and colonisation in PCR‐positive respiratory samples from immunocompromised patients.

**Methods:**

Adult patients with *P. jirovecii* detected by qPCR in respiratory samples (bronchoalveolar lavage (BAL), sputum and oral wash) collected between 2017 and 2023 were retrospectively enrolled in the study. Patients were classified as having PCP or *P. jirovecii* colonisation and Cq values were compared between the groups. Receiver‐operating characteristics (ROC) curve analyses were used to assess the performance of Cq values to distinguish between PCP and colonisation, and to establish Cq cut‐off values for the different sample types.

**Result:**

Of 520 included participants, 247 patients (47.5%) were classified as PCP and 273 (52.5%) as colonised. The median Cq value was significantly lower in the PCP group compared to colonised patients in BAL (33.0 vs. 36.6, *p* < 0.001) and sputum (33.4 vs. 36.0, *p* < 0.0001), yielding a ROC area under the curve of 0.75 and 0.73, respectively. Cq levels for oral wash did not differ between PCP and colonisation and lacked discriminatory power with a ROC AUC of 0.45. A Cq cut‐off level at 31 for BAL and sputum could predict PCP with a positive predictive value of > 85% while Cq < 38 provided a negative predictive value of 89% for BAL and 73% for sputum.

**Conclusion:**

Different Cq cut‐off values in BAL and sputum may support discrimination between PCP and colonisation and assist physicians in their clinical management of PCP.

## 1 Background

*Pneumocystis jirovecii* pneumonia (PCP) is a severe opportunistic infection caused by the fungus *Pneumocystis jirovecii* [1, 2, 3]. It predominantly affects immunocompromised individuals and is associated with high mortality rates [4, 5]. Historically, PCP was closely linked to HIV/AIDS and had a high prevalence during the peak of the HIV epidemic [1, 6]. However, widespread implementation of prophylactic treatment and antiretroviral therapy has significantly reduced the incidence of PCP in HIV‐positive populations. In contrast, PCP has increased in other immunocompromised groups, including individuals with haematological and solid malignancies, solid organ transplant (SOT) recipients and in patients with autoimmune disorders [4, 7, 8, 9, 10, 11].

The clinical diagnosis of PCP is based on a combination of clinical symptoms, radiological findings consistent with PCP, evidence of immunosuppression and microbiological identification of *P. jirovecii* in respiratory samples [12]. There is no gold standard for diagnosing PCP, and distinguishing PCP from other conditions, such as drug‐induced pneumonitis or alternative causes of acute respiratory distress syndrome, can be challenging in clinical practice. Additionally, *P. jirovecii* colonisation of the lower respiratory tract has been reported in approximately 20% of the general population, and in up to 69% in selected immunocompromised groups [2, 4, 13, 14]. The high prevalence of *P. jirovecii* colonisation further complicates the correct diagnosis of PCP. Treatment of PCP with high‐dose trimethoprim‐sulfamethoxazole (TMP/SMX) is effective but associated with significant adverse effects, including nephrotoxicity and bone marrow suppression. Conversely, untreated PCP can result in fatal outcomes for the patients [3, 15, 16, 17]. Consequently, accurately differentiating PCP from colonisation is essential for optimal patient management and for guiding decisions regarding treatment initiation.

Bronchoalveolar lavage (BAL) fluid is considered the gold standard specimen for the diagnosis of PCP, as it provides the most representative sample of the lower airways. However, BAL is an invasive procedure that may be challenging to perform in routine clinical settings [2, 12]. Alternative respiratory samples, such as sputum, induced sputum and oral wash, are less invasive but may yield lower diagnostic accuracy [1, 2, 18]. The reference method for a definitive PCP diagnosis in respiratory samples is by microscopic identification of *P. jirovecii* [12]. However, this method is user‐dependent, time‐consuming and has a limited sensitivity [1, 4, 6, 8, 12, 19], particularly in non‐HIV patients who typically have a lower *P. jirovecii* burden compared to HIV‐positive individuals [4, 20]. Since the 1990s, PCR‐based methods have been developed for diagnosing PCP. PCR has a high sensitivity for the detection of *P. jirovecii* in BAL fluid and sputum, and a relatively high sensitivity in oral wash samples [1, 2, 8, 18]. On the other hand, the high sensitivity of PCR introduces challenges in distinguishing between PCP and colonisation [4, 7]. Quantitative PCR (qPCR) has become a commonly used tool for *P. jirovecii* detection, providing the ability to assess the fungal load by measuring the quantification cycle (Cq), which indicates the number of amplification cycles required to detect the targeted DNA. Several studies have attempted to establish Cq cut‐off values to differentiate PCP from colonisation in PCR‐positive patients, but these efforts have primarily focused on BAL samples and a small number of patients [6, 10, 21]. In the present study, we aimed to assess the utility of qPCR for distinguishing PCP from *P. jirovecii* colonisation in a clinical routine setting using various respiratory sample types from PCR‐positive patients with diverse underlying conditions. Specifically, our goal was to establish Cq cut‐off values for BAL, sputum and oral wash that could discriminate PCP from colonisation with a sensitivity of 90% and a specificity of 80%.

## 2 Materials and Methods

### 2.1 Setting and Study Population

This is a retrospective study of patients positive for *P. jirovecii* in respiratory samples in Skåne, a region in southern Sweden. The study period spans from January 2017, when Cq values began to be registered, to March 2023. Patients were identified via the database at the Clinical Microbiology Department at Region Skåne, which serves all nine hospitals in the region and a population of 1,400,000 individuals.

All adult patients with a positive test result of *P. jirovecii* by PCR or microscopy in BAL/tracheal aspirates, sputum or oral wash samples were evaluated for inclusion in the study. Exclusion criteria were age < 18 years, missing Cq value, unavailable medical charts and patients classified with possible PCP according to the definition described below. Each patient could contribute more than one respiratory sample to the study, but only if they were of different respiratory materials (BAL, sputum or oral wash). Each respiratory sample type was analysed separately, and with this approach, we avoided the risk of bias due to sample weighting.

Clinical data, such as symptoms, medical history, radiology findings, laboratory test results and treatment were obtained from medical records. All data relating to *P. jirovecii* diagnostics, including Cq values from PCR, were provided by the clinical microbiology laboratory at Region Skåne. REDCap electronic data capture tools (Vanderbilt University, Nashville, Tennessee) were used for the collection and management of all study data.

### 2.2 PCP Definition

PCP diagnosis was classified based on modified criteria from the European Organisation for Research and Treatment of Cancer and the Mycoses Study Group's guidelines for PCP definitions in research (EORTC) [12]. These included findings of *P. jirovecii* in BAL, sputum or oral wash in combination with clinical symptoms, radiological findings, immunodeficiency, treatment for PCP and absence of an alternative diagnosis.

Clinical symptoms were defined as: Respiratory symptoms including cough, dyspnea or hypoxemia (at least 1 of 3) ± fever.

Radiological findings consisting of: Bilateral diffuse ground‐glass opacity, interstitial infiltrates on X‐ray or CT scan. Only lobar infiltrates were considered insufficient.

Immunodeficiency was defined as the EORTC criteria with the addition of prednisolone ≥ 15 mg/day for ≥ 4 weeks.

All participants were classified as having proven, probable or possible PCP or *P. jirovecii* colonisation according to the following definitions:

- Proven PCP: *P. jirovecii* visualised with microscopy in respiratory specimens, clinical and radiological findings and immunosuppression.
- Probable PCP: Positive *P. jirovecii*‐PCR together with symptoms, radiological findings and immunosuppression. No other diagnosis is more likely, and PCP treatment is given.
- Possible PCP: Positive *P. jirovecii*‐PCR with 2 of the following criteria: symptoms, radiological findings and/or immunosuppression. No other diagnosis is more likely and PCP treatment is given.
- Colonisation: Positive *P. jirovecii*‐PCR with only one criterion (symptoms, radiological findings or immunosuppression) OR positive *P. jirovecii*‐PCR together with 2 or 3 criteria (symptoms, radiological findings or immunosuppression), but other diagnoses are more likely and the patient did not receive PCP treatment.

In this study, patients with proven and probable PCP were regarded as PCP cases. Individuals with possible PCP were excluded from further analyses due to the uncertain *P. jirovecii* status.

### 2.3 Quantitative PCR

Respiratory samples were analysed for *P. jirovecii* DNA with qPCR as part of the clinical routine testing at the Clinical Microbiology Laboratory in Lund. This duplex qPCR targets the *P. jirovecii* large subunit of mitochondrial ribosomal RNA (mtLSU) and the beta‐tubulin gene, with primers described previously [22, 23] (Tables S1 and S2).

Highly mucous respiratory samples were diluted 1:2 in tris‐buffer and incubated with proteinase K (Qiagen, Venlo, The Netherlands) at 56°C, shaken at 500 rpm for a minimum of 30 min or until dissolved. Viscous samples were not pre‐treated. 200 μL of the sample was heated at 95°C for 15 min. DNA was extracted using EZ1 Advanced XL, with the EZ‐1 DNA Tissue Kit (both Qiagen, Venlo, The Netherlands) and an elution volume of 100 μL. 5 μL of DNA template was added to 20 μL of PCR master mix (SensiFAST Probe No‐ROX Kit, Meridian Bioscience, Cincinnati, Ohio, USA). Amplification was performed using CFX96 (Bio‐Rad, Hercules, California, USA), with an initial 5‐min heating of 95°C, followed by 45 cycles of 95°C for 10 s and 60°C for 30 s.

The mtLSU PCR is more sensitive than the beta‐tubulin PCR, in part owing to the multi‐copy nature of mitochondrial rRNA genes. When both genes were detected, the result was reported as ‘*P. jirovecii* DNA detected’ without further comments. When only mtLSU was detected, the result was reported as ‘weak positive’ with a comment that colonisation and PCP can be difficult to separate. This study evaluated Cq values of the mtLSU gene to distinguish between PCP and colonisation. Cq values were not visible to the clinicians at the time of the diagnostic work‐up.

### 2.4 Statistical Analysis

Statistical analyses were performed using SPSS, IBM SPSS Statistics (version 29.0.2.0 20). Continuous variables were described by median and interquartile range (IQR) and categorical values with frequency and percentage. Comparisons of Cq values between groups were made using the Mann–Whitney *U* test. Categorical variables were compared with the Chi‐square test. Uni‐ and multivariate logistic regression analyses were used to evaluate the effect of underlying immunodeficiencies on the association between Cq values and PCP. The Cq value was analysed as a continuous variable. The multivariate model was adjusted for HIV, lymphoma and other haematologic diseases. The level of significance was set to *p* < 0.05. GraphPad PRISM 10.3.1 (464) was used to create boxplots.

Sensitivity, specificity, positive and negative predictive values (PPV and NPV) were calculated based on Cq value thresholds identified in the ROC analyses. Youden's index was used to identify cut‐off levels with optimal sensitivity and specificity for each sample. Receiver‐operating characteristics (ROC) and area under the curve (AUC) analyses were used to assess the diagnostic performance of Cq values to distinguish between PCP and colonisation for different sample types.

## 3 Results

### 3.1 Patient Characteristics

**FIGURE 1:**
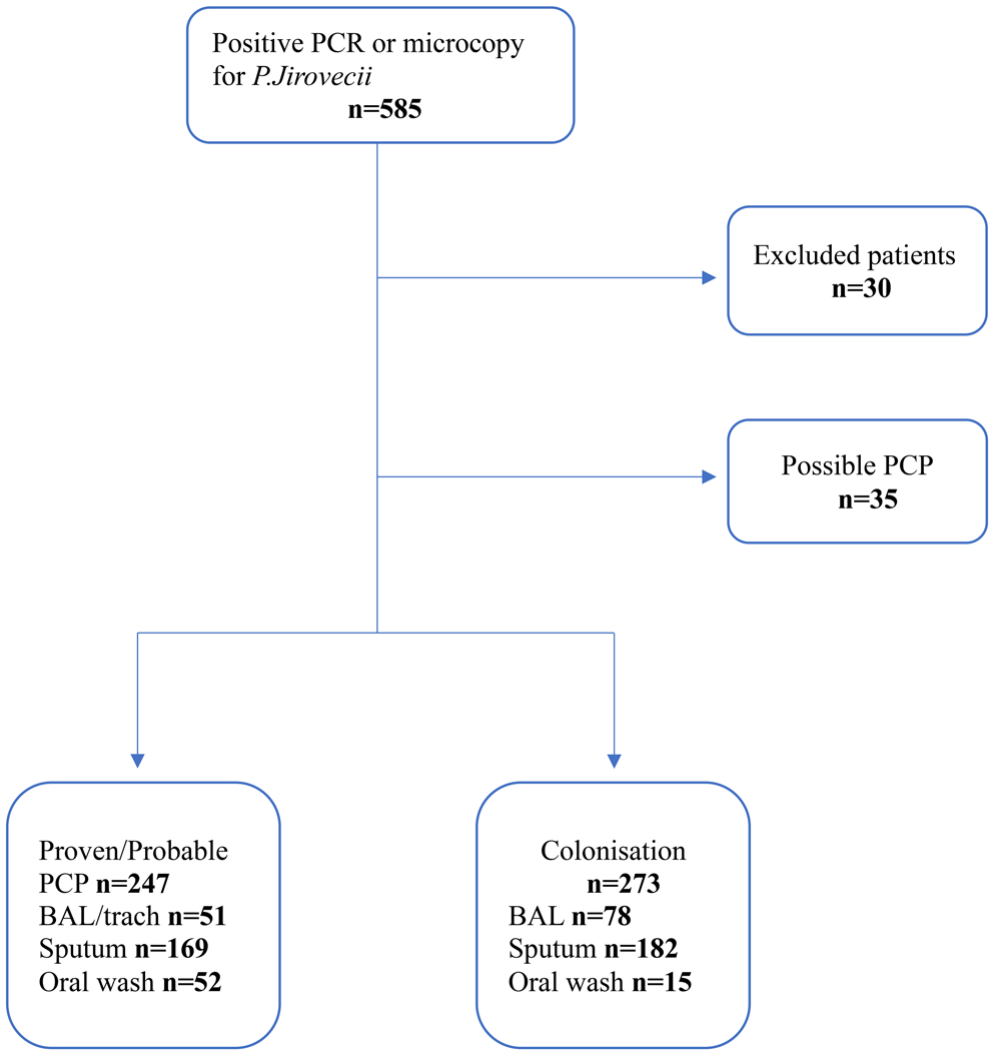
Flowchart for included patients and different sample types. Excluded patients: 7 age < 18 years, 7 no medical record, 8 missing Cq‐value and 8 NPH samples. Proven/Probable PCP: 25 extra materials, 12 sputum and 13 oral wash. Colonisation: 2 extra oral wash.

A total of 520 patients remained in the study after exclusion of non‐eligible patients (*n* = 30) and of the ‘possible PCP’ group (*n* = 35). Of the included participants, 247 patients (47.5%) were classified as having PCP, and 273 patients (52.5%) were considered colonised. Twenty‐seven patients contributed with more than one respiratory sample, collected from different specimen types. In the PCP group, 12 patients provided a sputum sample in addition to BAL, and 13 patients provided an oral wash sample in addition to BAL or sputum. In the colonised group, two patients provided both an oral wash sample and either a sputum or BAL sample (Figure 1).

Clinical and demographic data are presented in Table 1. The median age was 70 years in the PCP group versus 71 years in the colonisation group, and both groups showed a predominance of male patients. The most common immunocompromising conditions in the PCP group were solid tumours followed by haematological malignancies, rheumatic diseases and lymphomas. This deviated from the colonisation group, where solid tumours were followed by lung disease. Patients with HIV represented 3.7% of the total cohort. Cough was the most frequently reported clinical symptom in the PCP group, whereas dyspnea was more common in the colonisation group. The typical radiological pattern was absent in 34.8% of patients in the colonisation group, and all but one had an alternative, more likely diagnosis. Among participants with available 1,3‐ß‐d‐glucan (BDG) results, 98.7% tested positive for BDG in the PCP group, compared to 86.1% in the colonisation group (Table 1).

**TABLE 1:** Baseline characteristics.

	PCP, *n* = 247 (47.5%)	Colonization, *n* = 273 (52.5%)	*p*
**Demography**			
Age year: Median (IQR)	70 (60–77)	71 (63–77)	0.48
Male gender: *n* (%)	159 (64.4)	169 (61.9)	0.56
**Underlying diseases: *n* (%)**			
Solid tumours	86 (34.8)	67 (24.5)	0.01
Haematological diseases	42 (17)	30 (11)	0.047
Rheumatic diseases	38 (15.4)	43 (15.8)	0.91
Lymphomas	37 (15)	20 (7.3)	0.005
Lung diseases	22 (8.9)	53 (19.4)	< 0.001
HIV infection	14 (5.7)	5 (1.8)	0.02
SOT	12 (4.9)	12 (4.4)	0.80
**PCP criteria: *n* (%)**			
Host factor	247 (100)	189 (69.5)	< 0.001
Typical radiological pattern	247 (100)	178 (65.2)	< 0.001
Other diagnosis more likely	0 (0)	272 (99.6)	< 0.001
Clinical symptoms			
Fever (≥ 38°C)	177 (71.7)	154 (56.6)	< 0.001
Cough	168 (86.3)	186 (68.4)	0.07
Dyspnea	210 (85.0)	204 (74.7)	0.004
Hypoxemia	208 (84.6)	183 (67.0)	< 0.001
BDG+/BDG−	160/2 (98.7)	105/17 (86.1)	< 0.001
BDG‐data missing	85 (34.4)	151 (55.3)	< 0.001

Abbreviations: BDG, 1,3‐ß‐D‐glucan; BDG+, positive value, BDG−: Negative value; IQR, Interquartile range; PCP, *Pneumocystis jirovecii* pneumonia; SOT, Solid organ transplantation.

### 3.2 Patients With PCP Have Lower Cq Values Compared to Colonised Patients

**FIGURE 2:**
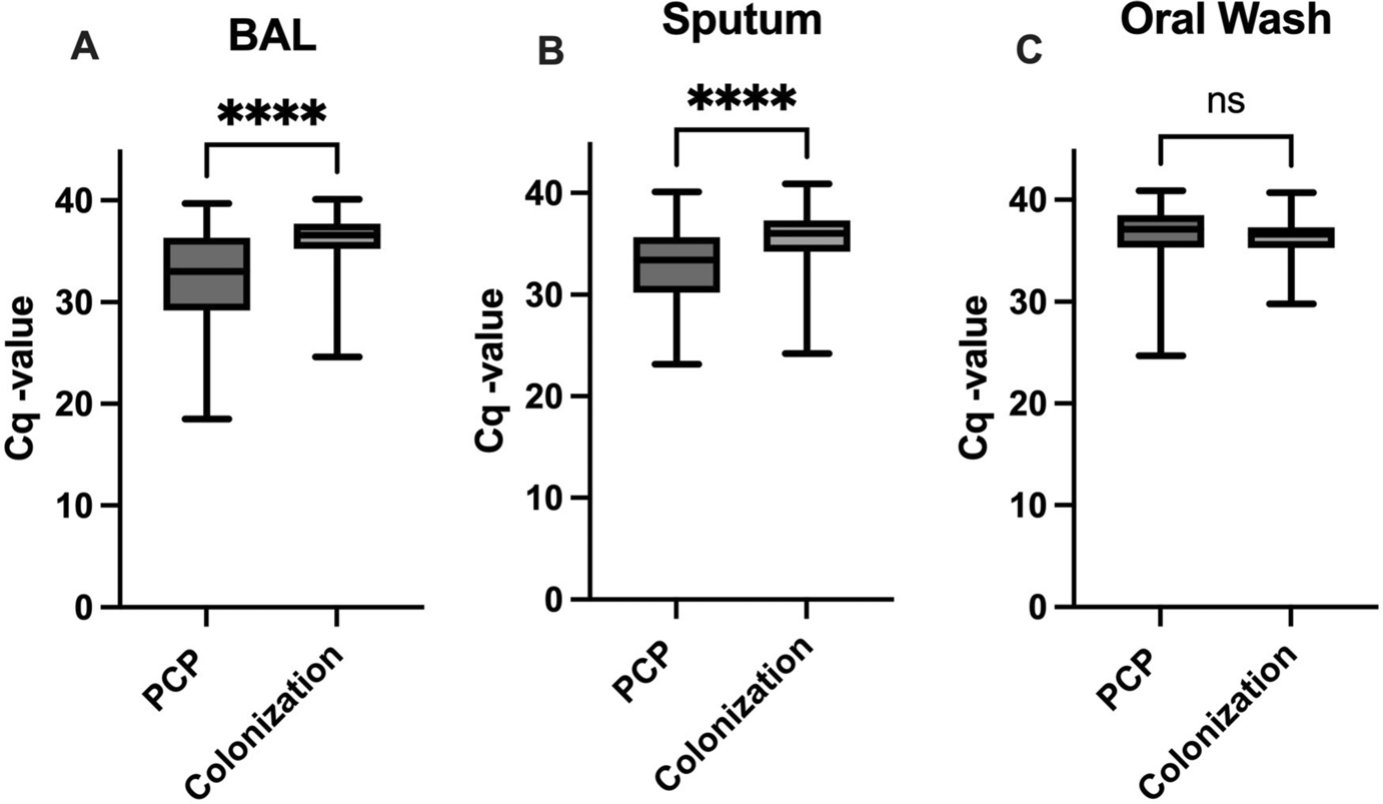
Boxplot showing median Cq‐values for PCP and colonisation in each sample type with minimum, maximum and IQR. (A) BAL, (B) sputum and (C) Oral wash. Mann–Whitney *U* test was used for comparison of the median, ****p < 0.0001. BAL, Bronchoalveolar lavage; Cq, Quantification cycle; ns, not significant.

The fungal load, quantified with PCR Cq values, was compared between the PCP and colonisation groups within each type of respiratory sample (Figure 2 and Table S3). Median Cq values were significantly lower in the PCP group compared to the colonisation group both in BAL 33.0 [IQR 29.2–36.3] versus 36.6 [35.3–37.7], (*p* < 0.001) and sputum 33.4 [30.2–35.6] versus 36.0 [34.2–37.3], (*p* < 0.001) (Figure 2A,B). No significant difference in Cq values was observed between the two groups for oral wash samples 37.1 [35.3–38.5] versus 36.6 [35.3–37.3], (*p* = 0.59) (Figure 2C). In a univariate logistic regression analysis, lower Cq values in BAL and sputum were significantly associated with PCP, with an odds ratio (OR) of 0.76 (95% CI 0.68–0.86, *p* < 0.001) and 0.76 (95% CI 0.71–0.83, *p* < 0.001), respectively. The corresponding OR for oral wash was 1.02 (95% CI 0.82–1.27, *p* = 0.86). A multivariate analysis adjusted for HIV, lymphoma and haematological diseases did not affect the estimates (Table S4).

Since PCP in individuals with HIV generally results in a higher *P. jirovecii* burden and a lower Cq value [10], we made a subgroup analysis after exclusion of 14 HIV patients (17 samples in the PCP group and 5 from the colonisation group). The median Cq values in BAL and sputum were higher after exclusion, but there was still a significant difference in Cq values between PCP and colonisation in both BAL and sputum (*p* < 0.001) (Table S5, and Figure S1).

### 3.3 Cq Values and Cut‐Offs to Distinguish PCP From Colonisation in Each Sample Type

**FIGURE 3:**
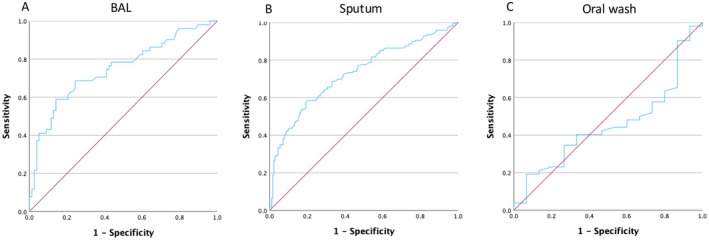
ROC curves for different sample types showing sensitivity and specificity for Cq cut‐off values to distinguish PCP from colonisation. (A) BAL, Area under the curve (AUC) 0.748 (95% CI 0.658–0.838). (B) Sputum, AUC 0.729 (95% CI 0.676–0.783). (C) Oral wash, AUC 0.454 (95% CI 0.299–0.609).

To assess the performance of Cq values to distinguish between PCP and colonisation, and to establish Cq cut‐offs which can identify PCP with a sensitivity of 90% and a specificity of 80%, ROC curve analyses were performed. Cq values in BAL and sputum were best at differentiating between PCP and colonisation with an area under the curve (AUC) of 0.75 (95% CI 0.66–0.84) and 0.73 (95% CI 0.68–0.78), respectively. Cq values in oral wash samples had an AUC of 0.45 (95% CI 0.30–0.61), indicating a lack of ability to distinguish between PCP and colonisation (Figure 3).

To determine Cq cut‐off values for distinguishing between PCP and colonisation, we applied Youden's index, which optimises the trade‐off between sensitivity and specificity. This analysis identified optimal cut‐off values of ≤ 33.9 for BAL, ≤ 34.0 for sputum and ≤ 34.7 for oral wash (Table 2 and Table S6). At these thresholds, all sample types identified PCP with a specificity > 80%, yielding positive predictive values (PPVs) of 73% for BAL and sputum, and 91% for oral wash. However, none of them fulfilled the aim of a sensitivity > 90% (Table 2). Choosing a lower Cq cut‐off level of < 31 increased the specificity to > 90% for BAL and sputum, but at the loss of sensitivity, 37.3% and 30.2%, respectively.

**TABLE 2:** Different Cq cut‐off values to distinguish PCP from colonization.

	Cut off	Sensitivity, % (95% CI)	Specificity, % (95% CI)	PPV, % (95% CI)	NPV, % (95% CI)
**BAL**					
	< 31	37.3 (24.1–51.9)	96.2 (89.2–99.2)	86 (66–95)	71 (65–74)
	≤ 33.9^a^	58.8 (44.2–72.4)	85.9 (76.2–92.7)	73 (60–83)	76 (69–82)
	< 38	94.1 (83.8–98.8)	21.8 (13.2–32.6)	44 (41–47)	89 (63–95)
**Sputum**					
	< 31	30.2 (23.4–37.7)	95.6 (91.5–98.1)	87 (76–93)	60 (57–62)
	≤ 34.0^a^	58.6 (50.8–66.1)	80.2 (73.7–85.7)	73 (67–79)	68 (63–72)
	< 38	91.7 (86.5–95.4)	18.7 (13.3–25.1)	51 (49–53)	73 (57–81)
**Oral wash**					
	≤ 34.7^a^	19.2 (9.6–32.5)	93.3 (68.1–99.8)	91 (58–99)	25 (22–29)

^a^ Youden's index.

To reach a sensitivity of > 90%, a higher cut‐off level was chosen. At Cq < 38, both BAL and sputum identified PCP with a sensitivity of > 90%, but with a low specificity of 21.8% and 18.7%, respectively. For oral wash, a Cq value of 39.5 gave a sensitivity of 90.4% and a specificity of 13.3%.

To assess how the HIV group affected our results, we did a subgroup ROC analysis after exclusion of HIV patients. Although the median Cq value was higher in the PCP group after exclusion of HIV patients (BAL median 33.8 [30.0–36.7] and sputum 33.6 [30.6–35.8]), the cut‐off for PCP with a specificity of > 80% was unchanged at Cq < 34 in both BAL and sputum (Table S7).

## 4 Discussion

This retrospective study demonstrates that quantification of *P. jirovecii* DNA may improve the diagnostic discrimination between PCP and colonisation in sputum and BAL samples. Although we were unable to find a single Cq cut‐off value that fulfilled our aim of 90% sensitivity and 80% specificity, we established separate Cq thresholds that independently achieved either the desired sensitivity or specificity. A Cq value < 31 in both BAL and sputum identified PCP with a PPV > 85%, while a Cq value ≥ 38 excluded PCP with a NPV of 89% for BAL and 73% for sputum. Notably, both BAL and sputum samples demonstrated very similar results, and a Cq threshold of < 31 can in our study be applied to both respiratory sample types. In agreement with our results, some previous studies have reported comparable cut‐offs between BAL and sputum, but most studies have only examined Cq values for BAL or small sample sizes of BAL and sputum [6, 7, 20, 21, 24]. While the established Cq thresholds may provide an additional clinically useful diagnostic tool, a diagnostic grey zone remains in which Cq values are not contributing to the distinction between PCP and colonisation. Therefore, clinical evaluation of symptoms, radiological findings and immunosuppressive treatment remains essential for accurate diagnosis.

In contrast to BAL and sputum samples, median Cq values for oral wash did not differ significantly between the PCP and colonisation group (Figure 2C). The ROC AUC for oral wash was 0.45, indicating a discriminatory performance worse than chance. Consequently, quantitative PCR did not improve the distinction between PCP and colonisation in this sample type.

Previous studies have identified varying Cq thresholds for the discrimination between PCP and colonisation [1, 6, 10]. However, differences in PCR protocols, DNA‐extraction methods, sample materials, patient populations and different definitions of PCP make it difficult to compare studies. PCR methods and *P. jirovecii* target genes can vary, and some previous studies analyse copies/ml instead of Cq values [4, 6, 18]. In general, Cq values have been shown to vary greatly between different laboratories [22], and it is therefore important that the local laboratory evaluates specific PCP cut‐offs applicable to their own diagnostic methods [8, 21]. Using copies/ml might provide a greater opportunity to compare results if the same gene is detected and a standard procedure is used for quantification [22]. Even so, there will still be variations between laboratories due to varying DNA extraction protocols and different BAL procedures.

This study included patients with different underlying diseases, which may reflect upon the results. For example, HIV patients are known to have a higher fungal burden, resulting in lower Cq values [1, 4, 10, 20]. Similarly, SOT patients have been reported to have a higher fungal load than other groups [6]. In a prospective study, it would be valuable to evaluate different cut‐offs for different immunocompromised groups. In the present study, the HIV group was too small (14 patients in the PCP group and five in the colonisation group) to evaluate Cq values in this group alone. Exclusion of HIV‐positive individuals resulted in a slightly higher median Cq value for the PCP group. However, there was still a significant difference between the PCP and colonisation groups for BAL and sputum, and the Cq cut‐off only changed slightly. Furthermore, the association between PCP and Cq values persisted after adjusting for HIV, lymphoma and haematological diseases in a multivariate logistic regression analysis (Table S4). Taken together, a general Cq cut‐off is a pragmatic approach in clinical routine testing and seems to be applicable on a mixed patient population. However, the results should be interpreted in the context of the patient's underlying condition.

The strengths of our study include a large cohort of 520 patients, contributing larger sample sizes compared to previous studies [1, 10, 19, 20, 21]. Moreover, we used a population‐based approach by including patients with positive PCR results at one single clinical microbiology laboratory that serves a whole region. Finally, most previous reports have evaluated Cq values for BAL whereas few have evaluated sputum samples and even less oral wash [6, 10, 21]. A strength of our study is the evaluation of all three sample types, which makes it possible to compare their diagnostic performance.

This study has several limitations, including the inherent challenge of accurately diagnosing PCP. This difficulty is not unique to our work, and previous studies have used different definitions or modified EORTC criteria to characterize PCP [3, 4, 6, 16, 20, 21, 25, 26]. In this study, we used modified EORTC criteria adjusted to our study setting. For example, the demonstration of *P. jirovecii* by immunofluorescent staining, which is required for a proven PCP diagnosis according to EORTC criteria [12], is not performed at our laboratory and samples are seldom sent to referral laboratories for analysis. Only one participant was classified as proven PCP in our cohort. We therefore included probable PCP, where the microbiological diagnosis was based on PCR results, in our definition of PCP cases. Additionally, BDG was not included in our diagnostic criteria for PCP due to missing data in 34.4% of the PCP group and 55.3% of the colonization group. Among participants with available BDG data, 98.7% of those in the PCP group tested positive (Table 1), suggesting accurate PCP classification. Although BDG is a helpful tool to predict the likelihood of PCP [15, 21], many other previous studies also lack BDG in their PCP assessment [21, 25]. Due to the diagnostic challenges of defining PCP, we chose to exclude patients classified as having ‘possible PCP’. Including this group would have increased the risk of misclassification, as they did not fully meet the EORTC criteria for a PCP diagnosis. Nevertheless, there is still a risk that colonised patients were misclassified as PCP and vice versa. Such misclassification would likely decrease, rather than enhance, the specificity and sensitivity of our data. Importantly, our PCP definition included two additional clinical assessment criteria, based on given PCP treatment and the evaluation of whether another diagnosis was more likely than PCP. We believe that this definition better reflects the distinction between PCP and colonization compared to EORTC criteria alone. All but one patient in the colonization group had another more likely diagnosis, indicating correct classification of colonised patients (Table 1).

Finally, Cq values were not available to clinicians. However, samples in which only the *mtLSU* gene was detected, but not the beta‐tubulin gene (which has a higher limit of detection), were reported as weakly positive. This information may have influenced clinicians' assessment of the patient and subsequent treatment decisions regarding PCP, thereby introducing a potential bias toward classifying patients as colonised with *P. jirovecii*. However, the risk of misclassifying true PCP cases as colonisation is considered low, as patients categorised as colonised did not receive PCP treatment during hospitalisation and untreated PCP would be expected to result in clinical deterioration.

In conclusion, PCP is a severe infection in both HIV patients and other immunodeficiency groups. The clinical discrimination between PCP and colonisation in patients with positive *P. jirovecii*‐DNA is challenging, and there is a need for improved diagnostic tools. Our study shows that a Cq value < 31 in BAL and sputum may distinguish between PCP and colonisation in a mixed immunocompromised patient population with a PPV of > 85%, while a Cq ≥ 38 indicates a low probability of PCP. These cut‐offs may help guide physicians in clinical decisions and improve the handling of this patient group.

## Author Contributions

**Sara Cederwall:** writing – original draft, software, formal analysis, methodology, investigation, conceptualization, validation, visualization, project administration, data curation. **Erik Ottander:** writing – review and editing, data curation. **David Björkhem:** writing – review and editing, data curation. **Karl Oldberg:** writing – review and editing, conceptualization, methodology. **Lisa I. Påhlman:** supervision, writing – review and editing, conceptualization, methodology, validation, resources, funding acquisition.

## Ethics Statement

The study was approved by the Swedish Ethical Review Authority (Reference number 2022–06955‐01). Informed consent was waived due to the retrospective study design.

## Conflicts of Interest

The authors declare no conflicts of interest.

## Supporting information


**Tables S1–S7:** myc70120‐sup‐0001‐TablesS1‐S7.docx.

## Data Availability

The data that support the findings of this study are available on request from the corresponding author. The data are not publicly available due to privacy or ethical restrictions.
