# Supplementary material for: Performance of Quantitative PCR to Distinguish Pneumocystis jirovecii Pneumonia From Colonisation in Immunocompromised Patients
Source: Mycoses. 2025 Oct 16;68(10):e70120. doi: 10.1111/myc.70120 (PMC12530012; doi:10.1111/myc.70120)
Supplement: Supplementary file 1 — Tables S1–S7: myc70120‐sup‐0001‐TablesS1‐S7.docx. [file MYC-68-e70120-s001.docx]

**Supplementary PCP-PCR**

**Table 1:** Designation of primers and probes, with their targets, final concentrations and product sizes. F = forward, R = reverse, P = probe.

| **Primer / probe** | **Target** | **Concentration in mix (µM)** | **Product (bp)** | **Reference** |
| --- | --- | --- | --- | --- |
| LSU-F | Mitochondrial large subunit rRNA (mtLSU) | 0.3 | 116 | 23 |
| LSU-R |  | 0.3 |  |  |
| LSU-P2 |  | 0.02 |  |  |
| β-tub 1120-F | β-tubulin | 0.1 | 132 | 22 |
| β-tub 1257-R |  | 0.1 |  |  |
| β-tub 1212T-P |  | 0.02 |  |  |
|  |  |  |  |  |

**Table 2**: Primer- and probe sequences for the mitochondrial large subunit rRNA (LSU) and β-tubulin (β-tub). F = forward, R = reverse, P = probe. The probes use the fluorophores FAM and HEX, and Black Hole Quencher 1 (BHQ1).

| **Primer / probe** | **Sequence 5’ – 3’** |
| --- | --- |
| LSU-F | TGC GAT AAG GTA GAT AGT CGA AAG G |
| LSU-R | AAG CCC ACT TCT TAA CTG TTT TAG ATA TCC A |
| LSU-P2 | FAM-ACA GCC CAG AAC AGT AAT TAA AGC TCC CCA-BHQ1 |
| β-tub 1120-F | CGG TCA TTG ACA GTT CCT GA |
| β-tub 1257-R | TTC AAC CTC CTT CAT GGA AAC AG |
| β-tub 1212T-P | HEX-TGT TGC AGC GAT TTT CCG CGG TA-BHQ1 |
|  |  |

**Table 3**: Respiratory materials divided into BAL, sputum and oral wash for PCP and Colonization and Cq values in each category.

|  | **BAL** | **Sputum** | **Oral wash** |
| --- | --- | --- | --- |
|  |  |  |  |
| **PCP** |  |  |  |
| No.materials | 51 | 169 | 52 |
| Median [IQR] | 33.0 [29.2–36.3] | 33.4 [30.2–35.6] | 37.1 [35.3–38.5] |
|  |  |  |  |
| **Colonization** |  |  |  |
| No.materials | 78 | 182 | 15 |
| Median [IQR] | 36.6 [35.3–37.7] | 36.0 [34.2–37.3] | 36.6 [35.3–37.3] |
|  |  |  |  |

**Table 4:** Logistic regression of Cq values correlated to PCP.

| **Variable**  **Background** | **Unadjusted**  **OR** | **95% CI** | **P-value** | **Adjusted#**  **OR** | **95% CI** | **P-value** |
| --- | --- | --- | --- | --- | --- | --- |
| **BAL** |  |  |  |  |  |  |
| Cq value | 0.764 | 0.678-0.861 | <0.001 | 0.780 | 0.686-0.886 | <0.001 |
| **Sputum** |  |  |  |  |  |  |
| Cq value | 0.765 | 0.707-0.829 | <0.001 | 0.762 | 0.703-0.826 | <0.001 |
| **Oral Wash** |  |  |  |  |  |  |
| Cq value | 1.019 | 0.819-1.269 | 0.864 | 1.016 | 0.806-1.282 | 0.892 |
|  |  |  |  |  |  |  |

**#**: Adjusted for HIV, lymphoma and hematological diseases.

**Table 5**: Respiratory materials divided into BAL, sputum and oral wash for PCP and Colonization. 14 HIV patients excluded in the PCP group (17 materials) and 5 patients (5 materials) in the colonization group. Cq values for each respiratory sample.

|  | **BAL** | **Sputum** | **Oral wash** |
| --- | --- | --- | --- |
| **PCP** |  |  |  |
| No.materials | 43 | 162 | 50 |
| Median[IQR] | 33.8 [30.0-36.7] | 33.6 [30.6–35.8] | 37.1 [35.3–36.5] |
|  |  |  |  |
| **Colonization** |  |  |  |
| No.materials | 76 | 180 | 14 |
| Median[IQR] | 36.6 [35.2–37.7] | 36.0 [34.3–37.3] | 36.5 [35.3–37.3] |
|  |  |  |  |

**Figure 1:** Boxplot to compare median Cq values for PCP and colonization. 14 HIV patients excluded in the PCP group and 5 patients in the colonization group. Mann-Whitney U test was used for comparison of the median, ****/*** : *p*<.0001. Cq: quantification cycle. BAL: bronchoalveolar lavage.

**Table 6:** Different Cq cut-off values to distinguish PCP from colonization. Youden’s index in bold.

|  | **Cut off value** | **Sensitivity** | **Specificity** | **PPV** | **NPV** |
| --- | --- | --- | --- | --- | --- |
| **BAL** | <30 | 31.4% | 96.2% | 85% | 69% |
|  | <31 | 37.3% | 96.2% | 86% | 71% |
|  | <32 | 43.1% | 91% | 73.3% | 71% |
|  | <33 | 49% | 87.2% | 72% | 71% |
|  | **</=33.9** | **58.8%** | **85.9%** | **73%** | **76%** |
|  | <35 | 62.7% | 76.9% | 63% | 77% |
|  | <36 | 70.6% | 62.8% | 54% | 76% |
|  | <37 | 84.3% | 35.9% | 47% | 80% |
|  | <38 | 94.1% | 21.8% | 44% | 89% |
|  |  |  |  |  |  |
| **Sputum** | <30 | 22.5% | 97.8% | 90% | 58% |
|  | <31 | 30.2% | 95.6% | 87% | 60% |
|  | <32 | 40.2% | 91.8% | 82% | 63% |
|  | <33 | 46.2% | 86.8% | 77% | 64% |
|  | **</=34.0** | **58.6%** | **80.2%** | **73%** | **68%** |
|  | <35 | 68.6% | 65.4% | 64% | 69% |
|  | <36 | 77.5% | 51.6% | 59% | 70% |
|  | <37 | 86.4% | 33% | 54% | 71% |
|  | <38 | 91.7% | 18.7% | 51% | 73% |
|  |  |  |  |  |  |
| **Oral wash** | </=34 | 13.5% | 93.3% | 88% | 24% |
|  | **</=34.7** | **19.2%** | **93.3%** | **91 %** | **25%** |
|  | <35 | 19.2% | 86.7% | 83% | 24% |
|  |  |  |  |  |  |

**Table 7**: Different Cq cut off values to distinguish PCP from colonization after exclusion of HIV patients. Youden’s index in bold.

|  | **Cut off value** | **Sensitivity** | **Specificity** |
| --- | --- | --- | --- |
| **BAL** |  |  |  |
|  | <31 | 30.2% | 96.1% |
|  | <32 | 34.9% | 90.8% |
|  | <33 | 39.5% | 86.8% |
|  | <34 | 51.2% | 85.9% |
|  | **</=35.3** | **62.8%** | **75.0%** |
|  | <36 | 65.1% | 63.2% |
|  | <37 | 81.4% | 35.5% |
|  | <38 | 93% | 22.4% |
| **Sputum** |  |  |  |
|  | <31 | 27.2% | 95.6% |
|  | <32 | 37.7% | 91.7% |
|  | <33 | 43.8% | 86.7% |
|  | **</=34.0** | **56.8%** | **80.6%** |
|  | <35 | 67.3% | 65.6% |
|  | <36 | 76.5%% | 51.9% |
|  | <37 | 85.8% | 32.8% |
|  | <38 | 91.4% | 18.3% |
| **Oral wash** |  |  |  |
|  | **</=34.7** | **20%** | **92.9%** |
|  |  |  |  |
